# Machine Learning Model for Mild Cognitive Impairment Stage Based on Gait and MRI Images

**DOI:** 10.3390/brainsci14050480

**Published:** 2024-05-09

**Authors:** Ingyu Park, Sang-Kyu Lee, Hui-Chul Choi, Moo-Eob Ahn, Ohk-Hyun Ryu, Daehun Jang, Unjoo Lee, Yeo Jin Kim

**Affiliations:** 1 Department of Electronic Engineering, Hallym University, Chuncheon 24252, Republic of Korea; qkrdlsrb3946@naver.com (I.P.); deahun123@naver.com (D.J.); 2 Department of Psychiatry, Hallym University-Chuncheon Sacred Heart Hospital, Hallym University College of Medicine, Chuncheon 24253, Republic of Korea; skmind@hallym.ac.kr; 3 Department of Neurology, Hallym University-Chuncheon Sacred Heart Hospital, Hallym University College of Medicine, Chuncheon 24253, Republic of Korea; dohchi@hallym.or.kr; 4 Department of Emergency Medicine, Hallym University-Chuncheon Sacred Heart Hospital, Hallym University College of Medicine, Chuncheon 24253, Republic of Korea; mooeob@gmail.com; 5 Division of Endocrinology and Metabolism, Department of Internal Medicine, Hallym University-Chuncheon Sacred Heart Hospital, Hallym University College of Medicine, Chuncheon 24253, Republic of Korea; ohryu30@gmail.com; 6 Division of Software, School of Information Science, Hallym University, Chuncheon 24252, Republic of Korea; 7 Department of Neurology, Kangdong Sacred Heart Hospital, Seoul 05355, Republic of Korea

**Keywords:** machine learning, mild cognitive impairment, gait, magnetic resonance imaging, convolutional neural network

## Abstract

In patients with mild cognitive impairment (MCI), a lower level of cognitive function is associated with a higher likelihood of progression to dementia. In addition, gait disturbances and structural changes on brain MRI scans reflect cognitive levels. Therefore, we aimed to classify MCI based on cognitive level using gait parameters and brain MRI data. Eighty patients diagnosed with MCI from three dementia centres in Gangwon-do, Korea, were recruited for this study. We defined MCI as a Clinical Dementia Rating global score of ≥0.5, with a memory domain score of ≥0.5. Patients were classified as early-stage or late-stage MCI based on their mini-mental status examination (MMSE) z-scores. We trained a machine learning model using gait and MRI data parameters. The convolutional neural network (CNN) resulted in the best classifier performance in separating late-stage MCI from early-stage MCI; its performance was maximised when feature patterns that included multimodal features (GAIT + white matter dataset) were used. The single support time was the strongest predictor. Machine learning that incorporated gait and white matter parameters achieved the highest accuracy in distinguishing between late-stage MCI and early-stage MCI.

## 1. Introduction

Mild cognitive impairment (MCI) is the prodromal stage of dementia [1]. While previous research has primarily focused on drug treatments for dementia, current efforts emphasise prevention and treatment before progressing to dementia, as this strategy is known to maintain a higher quality of life and yield more significant treatment effects [2]. Consequently, research into the characteristics of MCI has become increasingly active [3]. MCI is further categorised into early and late stages, based on the extent of cognitive impairment [4]. Patients with late-stage MCI have a higher likelihood of progressing to dementia [5].

It is known that MCI primarily affects cognitive function in the early stages, but as brain atrophy progresses and cognitive impairment worsens, it also affects motor function. Previous studies have reported motor function decline in patients with mild cognitive impairment. Additionally, changes in gait have been noted to become more pronounced as the degree of cognitive impairment worsens [6,7]. Previous research has shown that gait characteristics differ according to the MCI stage [8], thereby suggesting that gait characteristics can serve as indicators of MCI stages.

Efforts have been made to discover diagnostic biomarkers that were previously unidentified through traditional means using machine learning (ML) technology [9,10]. ML techniques for analysing brain imaging data have become prevalent in medical data analysis [11]. In particular, attempts have been made to distinguish between normal cognitive function and dementia or between normal cognitive function and MCI using ML applied to brain imaging data [12]. Machine learning can encompass various clinical data, not just MRI data. Previous research has utilised cognitive data, activity of daily living, and behavioural and psychological symptoms of dementia to differentiate between MCI and Alzheimer’s disease (AD) through machine learning [13], and diverse biomarkers and clinical data have been employed to predict the prognosis of MCI through machine learning [14]. Additionally, studies using physiological data from wearable devices have shown results in predicting cognitive function in MCI [15]. Furthermore, machine learning utilising gait information has been employed to distinguish types within MCI [16].

Distinguishing late-stage MCI, which has a higher likelihood of progressing to dementia, from early-stage MCI could also aid in predicting the prognosis of MCI and applying interventions. To explore a novel approach for differentiating between early-stage and late-stage MCI, we utilised machine learning with both MRI and clinical data, specifically focusing on gait data. We aimed to determine whether MRI findings or gait data were more effective in distinguishing between early-stage and late-stage MCI, and to identify specific parameters within MRI and gait data that differentiate between the two stages. Therefore, we conducted a classification of early-stage and late-stage MCI within the realm of MCI using both gait data and MRI imaging data.

## 2. Materials and Methods

### 2.1. Participants

We prospectively recruited 80 patients with MCI at Chuncheon Sacred Heart Hospital between October 2020 and April 2021. The inclusion criteria were as follows: (a) age 40–100 years; (b) patients with MCI who met the diagnostic criteria for minor neurocognitive disorder as determined by the Diagnostic and Statistical Manual of Mental Disorders Fifth Edition (DSM-V); (c) the absence of dementia according to physicians’ judgment; and (d) a Clinical Dementia Rating (CDR) global score of 0.5, with a memory domain score of ≥0.5. The exclusion criteria were as follows: (a) severe illness with an anticipated fatal outcome within 3 months; (b) a language barrier; (c) deafness or blindness; and (d) the lack of ability to provide informed consent. The patients were classified into two groups: (a) late-stage MCI, which showed performance on the MMSE of <1.5 standard deviations (SDs) below the normative mean; and (b) early-stage MCI, which showed performance on the MMSE of ≥1.5 SDs below the normative mean.

Written informed consent was obtained from each patient; the Institutional Review Board (IRB) of Chuncheon Sacred Heart Hospital approved the study protocol (IRB number: Chuncheon 2020-09-005; approved on 12 October 2020).

### 2.2. Clinical Assessment

We recorded the patients’ demographics (age, sex, and years of education) and body composition measures (height, weight, and waist circumference). We also determined their blood pressure and conducted specific blood tests (i.e., fasting glucose and total cholesterol). The patients completed the depression (Short form of Geriatric Depression Scale, SGDS), anxiety (Korean Geriatric Anxiety Inventory, K-GAI), and quality of life (Geriatric Quality of Life Dementia, GQOL-D) scales, as well as the Korean National Health and Nutrition Examination Survey (KNHANES). Additional medical comorbidities were checked during the in-clinic interviews.

### 2.3. Gait Assessment

The GAITRite^®^ instrumentation (CIR systems Inc., Havertown, PA, USA) consists of an electronic walkway of 5.6 m in length and 0.9 m in width. Each patient was instructed to walk at a normal pace without a gait aid on the walkway. Each patient walked on the GAITRite^®^ pad in a single pass. The study coordinator observed the gait of each patient without interfering with it. The present analysis focused on individual spatial (stride and step lengths), temporal (gait speed, step count, cadence, and stance time), and spatiotemporal (cadence) parameters.

### 2.4. Datasets for Machine Learning

Gait characteristics and MRI features were obtained at the patients’ baseline visits. For each subject, the measurements extracted from the gait and MRI data were combined as follows: (a) Gait dataset (unimodal dataset): this contained the GAITRITE-derived measures (74 features per subject); (b) Gray matter dataset: this contained the gray matter area among the MRI-derived matrix (476 features per subject); (c) White matter dataset: this contained the white matter area among the MRI-derived matrix (27 features per subject); (d) MRI dataset (multimodal dataset): this contained the entire MRI-derived matrix and was obtained by combining (b) and (c) into a single dataset, which resulted in 503 features per subject; (e) Gait + gray matter dataset (multimodal dataset): this was obtained by unifying (a) and (b) into a single dataset, resulting in 550 features per subject; (f) Gait + white matter dataset (multimodal dataset): this was obtained by combining (a) and (c) into a single dataset, resulting in 101 features per subject; (g) Gait + MRI dataset (multimodal dataset): this was obtained by unifying (a) and (d) into a single dataset, resulting in 577 features per subject.

### 2.5. MR Imaging Techniques

Standardised T2-, fluid-attenuated inversion recovery (FLAIR)-, and three-dimensional (3D) T1-weighted images were acquired from all eligible participants at the Chuncheon Sacred Heart Hospital using the same 3.0T MRI scanner (Siemens Skyra). We acquired 3D T1-weighted structural brain images using a Magnetisation Prepared Rapid Acquisition Gradient Echo (MPRAGE) sequence with the following parameters: sagittal slice thickness of 1.0 mm, no gap, repetition time (TR) of 2300.0 msec, echo time (TE) of 2.98 msec, flip angle of 9°, inversion time (TI) of 900 msec, and imaging matrix size of 256 × 240 × 176. The following parameters were used for the FLAIR images: (a) axial slice thickness of 2 mm; (b) no gap; (c) TR of 11,000 msec; (d) TE of 125 msec; (e) flip angle of 90°; and (f) matrix size of 512 × 512 pixels. All axial sections were acquired parallel to the anterior and posterior commissures.

### 2.6. Gray Matter Measurements

For this study, 3D Slicer (http://www.slicer.org, accessed on 28 April 2024; Surgical Planning Laboratory, Harvard University, Boston, MA, USA, version 4.11) and FreeSurfer (http://www.freesurfer.net, accessed on 28 April 2024; MIT Health Sciences & Technology, and Massachusetts General Hospital, USA, version 7.1.1) software were used to measure the total volume, mean and SD of thickness, mean curvature, Gaussian curvature, folding index, and curvature index of the gray matter for each subcortical region from the 3D T1 image data in Digital Imaging and Communications in Medicine (DICOM) format. The DICOM files were converted into Neuroimaging Informatics Technology Initiative (NifTI) format using 3D Slicer and then reconstructed into a two-dimensional (2D) cortical surface using the recon-all function of FreeSurfer [17,18]. The reconstruction involved several steps of the recon-all function, including motion correction, skull stripping, normalisation and transformation, white and gray matter segmentation, averaging and smoothing, parcellation of subcortical regions, and measurement of parcellation statistics. The results of the parcellation statistics provided the values of the gray matter for each of the 34 subcortical regions per hemisphere.

### 2.7. White Matter Hyperintensity Measurements

Brain Intensity AbNormality Classification Algorithm (BIANCA) software (BIANCA, FSL 6.0.5) in FSL (FMRIB Software Library) was used for the segmentation and quantification of white matter hyperintensities (WMHs) in MRI images for each subject. The segmentation and quantification of WMHs include pipelined processing steps using tools in FSL. FLAIR MRI images were used as the standard template with six degrees of freedom. Then, a WMH classification model was generated using the BIANCA algorithm, in which the datasets of the MICCAI WMH segmentation challenge (http://wmh.isi.uu.nl/, accessed on 28 April 2024) were used as training sets and the following non-default options were used in the training: the location of training points = no border and the number of training points = 2000 lesion points and 10,000 non-lesion points.

The features of WMHs were estimated using the Nilearn library, a Python package (python 3.8.3) that facilitates the use of advanced machine learning techniques to analyse data acquired by MRI. A total of 27 WMH features were estimated; these were total WMH volume, total periventricular WMH, total deep WMH, total WMH volumes of the right and left four lobes, periventricular WMH volumes of the right and left four lobes, and deep WMH volumes of the right and left four lobes. Periventricular WMH volume was defined as the regional WMH volume within 10 mm of the edge of the ventricle in each axial slice; deep WMH volume was defined as the regional WMH volume outside 10 mm of the edge of the ventricle in each axial slice.

### 2.8. Deep Learning Analysis

Three unsupervised ML algorithms for feature reduction were trained using features from each of the seven datasets: independent component analysis (ICA), principal component analysis (PCA), and random projection (RP). We considered 20, 40, and 60 as the numbers of reduced features. For each of the seven datasets, three supervised ML algorithms for classification were trained with reduced features corresponding to each number (platform): support vector machine (SVM), random forest (RF), and convolutional neural network (CNN). To evaluate the performance of the model architectures for the predictive classification and diagnosis of MCIs, SVM, RF, and CNN were constructed using various numbers of features reduced from three unsupervised ML algorithms. The CNN architecture used for reducing feature dimensions presented in Supplementary Table S1. The data were divided into training (80%) and test (20%) datasets. The training performance of the different ML models was evaluated using a 10-fold cross-validation; this process was repeated 10 times. Hyperparameter tuning was automatically performed by testing 60 different values of each hyperparameter.

A total of 27 combinations of MLs (9 combinations of 1 of the 3 MLs for classification combined with 1 of the 3 MLs for feature reduction) for each of the 3 different numbers (20, 40, and 60) of reduced features were tested, optimised using 60 hyperparameter variations, and evaluated using 10-fold cross-validation. In the testing phase, the prediction performance of each combination of ML models was evaluated using parameters, including the area under the receiver operating characteristic curve (AUC), accuracy (ACC), recall, precision, and F1 score. The entire process was repeated for the seven datasets: Gait, Gray matter, White matter, MRI, Gait + gray matter, Gait + white matter, and Gait + MRI. Performances of Gait dataset presented in Supplementary Table S2. Performances of MRI dataset presented in Supplementary Table S3. And performances of Gait + MRI dataset presented in Supplementary Table S4. The computing machine is used for timestamping runs on Ubuntu 18.04 and is equipped with an Intel Core i9-9820X CPU (10 cores, Intel^®^ Xeon^®^ Silver 4210, @2.20 Ghz, 128 GB RAM), 64 GB of memory, and an NVIDIA GTX 1080 Ti GPU (NVIDIA GeForce RTX 3090 24 GB).

### 2.9. Statistical Analysis

Baseline characteristics based on the data are presented as mean and standard deviation (SD) for continuous variables and percentages for categorical variables. Differences between the early-stage MCI and late-stage MCI groups were confirmed using Student’s *t*-test for continuous variables and the chi-square test for categorical variables. Statistical analyses were conducted using the SPSS version 27 software (SPSS Inc., Chicago, IL, USA).

## 3. Results

### 3.1. Baseline Characteristics

The detailed demographic and clinical characteristics of the participants are presented in Table 1. The early-stage MCI group exhibited a higher proportion of females compared to the late-stage MCI group. Years of education were shorter in the early-stage MCI group than in the late-stage MCI group. Additionally, the early-stage MCI group had a higher number of comorbidities than the late-stage MCI group. Body mass index (BMI) was higher in the early-stage MCI group than in the late-stage MCI group.

### 3.2. Classification Results

The machine learning classification of the participants is presented in Table 2. Three machine learning algorithms (SVM, RF, and CNN) were employed to identify the optimal feature pattern for distinguishing late-stage MCI from early-stage MCI. In the Gait dataset, the CNN demonstrated superior classification performance, achieving an AUC of 98% and an ACC of 99%. For the MRI dataset, the CNN also exhibited the best classification performance, with an AUC of 94% and an ACC of 96%. In the Gait + white matter dataset, the CNN displayed the highest classification performance, yielding an AUC of 96% and an ACC of 97%.

### 3.3. Top 10 Features of Discriminating between Late- and Early-Stage MCI

The top 10 features of discriminating late-stage MCI from early-stage MCI are presented in Table 3. Three different machine learning algorithms (ICA, PCA, and RP) were utilised for feature reduction. In the Gait dataset, the SD of the stride velocity of the right leg was the strongest predictor. Additionally, the stride length SD of the left leg and stance percentage of the cycle of the right leg were among the top predictors. The next most significant predictors were the swing time SD of the right leg, step time differential of the left leg, and stance time of the left leg. For the MRI dataset, the left fusiform thickness was the strongest predictor. The right inferior parietal and right supramarginal thicknesses were also among the top predictors. Following this, predictors such as right middle temporal, left supramarginal, right fusiform, and left inferior parietal thicknesses were identified. The single support time was the strongest predictor of classification using the Gait + white matter dataset. The total WMH volume of the right parietal area and single support percentage of the right leg cycle were among the top predictors. The next top predictors included the cycle time of the right leg and SD of the step length of the right leg.

Utilising the Gait dataset for machine learning with the CNN algorithm, the best performance was observed in distinguishing between early-stage and late-stage MCI. Among the top 10 features that distinguish late-stage MCI from early-stage MCI are stride velocity standard deviation of the right leg, stride length standard deviation of the left leg, stance percentage of cycle of the right leg, swing time standard deviation of the right leg, step time differential, stance time of the left leg, step time of the left leg, double support unload time of the left leg, stride length standard deviation of the right leg, and double support time standard deviation of the left leg.

## 4. Discussion

In our research, among several datasets, the model with the highest accuracy included only the gait datasets. Gait characteristics are closely related to cognitive impairment. In previous studies, patients with MCI exhibited a greater reduction in motor function than those without cognitive impairment [19]. Moreover, these results suggested that motor decline can manifest before the onset of MCI [20]. Decreased memory and executive functions are associated with gait impairment [21]. Therefore, gait disturbance can potentially serve as a more accurate indicator for differentiating the levels of cognitive impairment within MCI compared to brain MRI, which reflects structural changes.

Although the accuracy was slightly lower than that of the Gait dataset, the MRI dataset also exhibited a high accuracy of 96% in distinguishing early- from late-stage MCI. However, when distinguishing between gray and white matter, the accuracy for gray matter was notably high at 94%, whereas the accuracy for white matter dropped to 86%. This study exhibited better performance compared to previous research in the field. Table 4 demonstrates the performance of machine learning models utilised in patients with mild cognitive impairment in prior studies.

Cognitive function is partly a consequence of neuronal loss in the gray matter, thus making its volume highly relevant to cognitive function [22]. AD is characterised by cognitive impairment and a loss of gray matter volume [23]; therefore, it is expected that the pattern of gray matter loss will differ depending on the cognitive function stage of MCI [24]. White matter primarily consists of fibres through which neurones transmit signals [25]; this may explain why changes in the white matter are less relevant to cognitive function than changes in the gray matter. Both white and gray matter changes contributed to distinguishing between elderly individuals with normal cognition and those with MCI [26,27]. However, when it comes to reflecting the degree of cognitive function in MCI, gray matter atrophy was found to be a more significant factor [28].

Interestingly, despite our results, using the Gait + white matter datasets instead of the Gait + gray matter datasets resulted in higher accuracy for machine learning in distinguishing between early- and late-stage MCI. When gait parameters were included in the analysis, it was observed that white matter contributed to improved accuracy, whereas gray matter did not provide any assistance and further decreased accuracy. This is contrary to the results obtained using only MRI data. While the classification accuracy decreased when using only white matter information, it suddenly increased when gait data from a clinical dataset were included. This suggests that although white matter changes may not be directly correlated with cognitive impairment, they can serve as indicators of the degree of cognitive impairment. In previous studies, there was no difference in the overall white matter volume change between early- and late-stage MCI; however, there were differences in the specific regions of white matter change [29]. Since white matter changes do not directly cause functional abnormalities similar to gray matter changes, [30] using only the structural information of white matter changes makes it difficult to distinguish early- from late-stage MCI. However, our study indicates that when combined with data reflecting functions such as gait, the characteristics of white matter changes can be used as a better indicator to differentiate between the two forms.

Gait variability is recognised as a reliable indicator of cognitive function, which could explain why SD emerged as the top feature associated with late-stage MCI used in machine learning with gait data in our study. Reports have suggested that gait variability is a better predictor of cognitive decline in healthy elderly individuals and in patients with MCI [31,32]. Previous research has also demonstrated associations between double support phase variability and executive function, processing speed, and visuospatial ability, as well as a correlation between swing time standard deviation and global cognitive function, memory, attention, language, and visuospatial function [33]. These findings from prior studies may provide a basis for the selection of stride velocity SD or stride length SD as top features for determining the level of cognitive function within MCI in our research. Furthermore, in our study, the top feature distinguishing the Gait dataset corresponded mainly to the temporal parameters [33]. In a previous study, gait parameters such as step length and stride length, which are spatial parameters, distinguished the elderly with dementia and MCI from healthy individuals [6]. However, in elderly individuals with no cognitive impairment, executive function and processing speed had a stronger association with temporal parameters than with spatial parameters [34]. Furthermore, temporal parameters were better at distinguishing between non-MCI elderly groups and MCI groups compared to spatial parameters in previous research [32]. Considering that temporal parameters were predominantly included as the top features in our research results, it may be speculated that temporal parameters may better reflect the degree of cognitive impairment in MCI.

The top features used in machine learning with MRI data were consistent with the top 10 features used in machine learning with gray matter data alone. This suggested that in distinguishing the degree of cognitive impairment, gray matter characteristics were more relevant than gait characteristics. In particular, the fact that gray matter thickness, among other gray matter parameters, emerged as a top feature indicated that the degree of cognitive impairment in MCI was most significantly reflected by gray matter thickness. Among the top 10 features, the thicknesses of regions related to the temporal and parietal lobes, such as the fusiform gyrus, inferior parietal lobule, and supramarginal gyrus, were predominantly included. The fusiform gyrus plays a significant role in visual cognition; visual cognitive deficits are commonly reported in patients with AD [35]. Previous functional MRI studies have reported altered functional connectivity of the fusiform gyrus in patients with MCI as compared to subjects without cognitive impairment [35]. Given that the fusiform gyrus connects various regions in the occipital and temporal lobes and exhibits active connections with multiple areas, it may be one of the first areas to reflect cognitive function within MCI through its cortical thickness. The inferior parietal lobule, which consists of the supramarginal gyrus and angular gyrus, is a specific neuroimaging marker for predicting the conversion from MCI to AD [36]. Among the regions within the inferior parietal lobule, the supramarginal gyrus plays a crucial role in short-term memory storage and rehearsal [37]. It has also been reported in previous studies as an area that distinguishes MCI from normal cognitive function [38].

The top 10 features used in machine learning with the Gait + WMH dataset were single support time and white matter hyperintensity in the right parietal area. A previous study reported that white matter changes in the frontal area differed between patients with early-stage MCI and those with late-stage MCI [29]. However, in our study, using machine learning to distinguish between early-stage MCI and late-stage MCI, only white matter changes in the parietal area were included as distinguishing features. Frontal and parietal WMHs were associated with frontal glucose metabolism [39]. Therefore, it is likely that parietal WMHs also play a role in discriminating between the MCI cognitive levels. Moreover, executive function appeared to be influenced more by white matter changes and frontal glucose metabolism than by cortical atrophy [39]. Executive functions are also associated with gait disturbances [40]. Therefore, the improved classification of early- and late-stage MCI, when the data include both gait parameters and white matter changes, may be attributed to the differences in their respective executive function.

### Limitations

First, we did not classify the patients with mild amnestic cognitive impairment. Therefore, we cannot exclude the possibility that participants with a degenerative pathology related to parkinsonism or vascular damage may have been present. As our study targeted a heterogeneous population of patients with MCI, caution is required when interpreting the results. Secondly, musculoskeletal problems affecting gait disturbances were not considered. Third, because this was a cross-sectional study, it was difficult to establish causal relationships.

## 5. Conclusions

In this study, we classified patients with MCI into early- and late-stage MCI groups using gait characteristics and MRI features. Importantly, we discovered that gait characteristics were sufficiently accurate to distinguish between early-stage MCI and late-stage MCI. This implies that gait features can serve as markers for detecting the progression of cognitive impairment even within the MCI population.

## Figures and Tables

**Table 1:** Demographic and clinical characteristics.

	Total (n = 80)	Early-Stage MCI (n = 53)	Late-Stage MCI (n = 27)	*p* Value
Mean age	74.6 ± 5.74	74.6 ± 5.33	74.6 ± 6.58	0.950
Gender, female number (%)	58 (72.5)	43 (81.1)	15 (55.6)	0.020
Years of education	6.88 ± 3.980	5.94 ± 4.069	8.72 ± 3.114	0.003
Number of comorbidities	3.7 ± 1.70	3.9 ± 1.61	3.1 ± 1.77	0.047
Depression scale	4.9 ± 4.09	4.9 ± 4.24	5.1 ± 3.83	0.833
Anxiety scale	6.8 ± 6.52	7.1 ± 6.87	6.3 ± 5.86	0.600
QOL scale	34.4 ± 8.67	33.3 ± 8.39	36.5 ± 9.00	0.124
Height	156.1 ± 8.11	155.4 ± 7.98	157.7 ± 8.30	0.232
Weight	61.0 ± 10.92	61.9 ± 11.47	59.3 ± 9.72	0.324
BMI, mean (SD)	25.0 ± 3.70	25.6 ± 3.84	23.8 ± 3.17	0.045
Waist circumference (cm)	87.8 ± 10.12	87.6 ± 10.39	88.1 ± 9.75	0.825
Systolic BP (mmHg)	128.6 ± 18.27	130.0 ± 18.44	126.0 ± 17.97	0.349
Diastolic BP (mmHg)	77.0 ± 9.82	77.4 ± 9.41	76.1 ± 10.73	0.606
Fasting glucose (mg/dL)	108.8 ± 26.28	111.3 ± 28.44	104.0 ± 21.08	0.240
Total cholesterol (mg/dL)	162.4 ± 32.54	34.0 ± 4.67	30.1 ± 5.79	0.913

The data are mean ± SD unless otherwise indicated a Number (%). Abbreviations: MCI, mild cognitive impairment; QOL, quality of life; BMI, body mass index; BP, blood pressure; SD, standard deviation.

**Table 2:** Machine learning classification of patients with MCI.

Group	Algorithm for Classification	Algorithm for Feature Reduction	No. of Features Reduced	AUC	ACC	Recall	Precision	F1
Gait	CNN	RP	40	0.98 ± 0.04	0.99 ± 0.04	0.99 ± 0.03	0.99 ± 0.03	0.99 ± 0.04
Gray matter	CNN	PCA	20	0.92 ± 0.08	0.94 ± 0.07	0.94 ± 0.10	0.97 ± 0.05	0.95 ± 0.06
White matter	CNN	PCA	20	0.83 ± 0.14	0.86 ± 0.13	0.88 ± 0.11	0.96 ± 0.07	0.89 ± 0.11
MRI	CNN	PCA	20	0.94 ± 0.10	0.96 ± 0.08	0.95 ± 0.09	0.99 ± 0.03	0.97 ± 0.05
Gait + gray matter	CNN	PCA	40	0.95 ± 0.08	0.96 ± 0.08	0.97 ± 0.06	0.96 ± 0.08	0.96 ± 0.06
Gait + white matter	CNN	RP	60	0.96 ± 0.07	0.97 ± 0.06	0.97 ± 0.06	0.98 ± 0.04	0.98 ± 0.05
Gait + MRI	CNN	RP	60	0.94 ± 0.10	0.95 ± 0.10	0.95 ± 0.08	0.98 ± 0.04	0.96 ± 0.07

Abbreviations: AUC, area under the receiver operating characteristic curve; ACC, accuracy.

**Table 3:** Top 10 features associated with late-stage MCI (PCA component = 40).

GAIT Data	MRI Data	GAIT + WMH Data
Stride Velocity SD R	ThickAvg_L.fusiform	Single Support Time (sec) L
Stride Length SD L	ThickAvg_R.inferiorparietal	Single Support Time (sec) R
Stance % of Cycle R	ThickAvg_R.supramarginal	R parietal total WMHs
Swing Time SD R	ThickAvg_R.middletemporal	Single Support % Cycle R
Step Time Differential	ThickAvg_L.supramarginal	Cycle Time (sec) R
Stance Time (sec) L	ThickAvg_R.fusiform	Step Length SD R
Step Time (sec) L	ThickAvg_L.inferiorparietal	R parietal deep WMHs
Double Support Unload Time L	ThickAvg_L.precentral	Support Base SD R
Stride Length SD R	ThickAvg_R.precentral	Heel Off/On SD R
Double Support Time SD L	ThickAvg_L.lateralorbitofrontal	Toe In/Out R

Abbreviations: PCA, principal component analysis; SD, standard deviation; R, right; L, left; ThickAvg, mean thickness of gray matter.

**Table 4:** Previous studies using machine learning model in patients with mild cognitive impairment.

Reference	Algorithm	Feature Selection	Objective	Participants	Outcomes
Lin et al. [7]	RF	29 gene biomarkers	To predict stable MCI patients	195 normal, 271 MCI, and 112 AD	AUC of cross-validation and test dataset was 0.841 and 0.775, respectively
Lu et al. [11]	XGboost, Bayers, SVM, and LR	ADL, BPSD, and cognitive function	Differentiation of AD from MCI	458 AD and MCI	XGBoost with Precision was 0.82, Bayes with Precision was 0.75, SVM with Precision was 0.78, and LR with Precision was 0.81
Adelson et al. [12]	XGboost, KNN, MLP, and LR	Demographics, family medical history, comorbidities, and neuropsychiatric assessments	Identification of risk of progressing from MCI to AD	493 MCI	XGBoost with AUC at 12 months was 0.857, at 24 months, it was 0.980, and at 48 months, it was 0.975
Rykov et al. [13]	ElasticNet, RF, and XGBoost	106 digital physiological features	To predict cognitive function	30 MCI	RF with Pearson r was 0.61 in the individual-based cross-validation, whereas RF with Pearson r was 0.77 in the interval-based cross-validation
Chen et al. [14]	SVM	Gait analysis system to perform walk, time up and go, and jump test	To predict different types of MCI	34 PD MCI; 47 non-PD MCI	Accuracy was 91.67% and AUC was 0.9143 with polynomial kernel function

Abbreviations: RF, random forest; XGBoost, extreme gradient boosting; SVM, support vector machine; LR, logistic regression; KNN, k-nearest neighbour; MLP, multi-layer perceptron; ElasticNet, elastic net regression; ADL, activity of daily living; BPSD, behavioural and psychological symptoms of dementia; MCI, mild cognitive impairment; AD, Alzheimer’s disease; PD, Parkinson’s disease; AUC, area under the receiver operating characteristic curve.

## Data Availability

The data that support the findings of this study are available on request from the corresponding author. The data are not publicly available due to privacy or ethical restrictions.

## Supplementary Materials

The following supporting information can be downloaded at: https://www.mdpi.com/article/10.3390/brainsci14050480/s1, Supplementary Table S1. The CNN architecture used for N (=20, 40, or 60) reduced feature dimensions in this study. Supplementary Table S2. Performances of Gait dataset. Supplementary Table S3. Performances of MRI dataset. Supplementary Table S4. Performances of Gait + MRI dataset.

## References

[1] Petersen R.C. (2004). Mild cognitive impairment as a diagnostic entity. J. Intern. Med..

[2] Livingston G., Huntley J., Sommerlad A., Ames D., Ballard C., Banerjee S., Brayne C., Burns A., Cohen-Mansfield J., Cooper C. (2020). Dementia prevention, intervention, and care: 2020 report of the Lancet Commission. Lancet.

[3] Pandya S.Y., Clem M.A., Silva L.M., Woon F.L. (2016). Does mild cognitive impairment always lead to dementia? A review. J. Neurol. Sci..

[4] Aisen P.S., Petersen R.C., Donohue M.C., Gamst A., Raman R., Thomas R.G., Walter S., Trojanowski J.Q., Shaw L.M., Beckett L.A. (2010). Clinical Core of the Alzheimer’s Disease Neuroimaging Initiative: Progress and plans. Alzheimer’s Dement..

[5] Jessen F., Wolfsgruber S., Wiese B., Bickel H., Mosch E., Kaduszkiewicz H., Pentzek M., Riedel-Heller S.G., Luck T., Fuchs A. (2014). AD dementia risk in late MCI, in early MCI, and in subjective memory impairment. Alzheimer’s Dement..

[6] Bovonsunthonchai S., Vachalathiti R., Hiengkaew V., Bryant M.S., Richards J., Senanarong V. (2022). Quantitative gait analysis in mild cognitive impairment, dementia, and cognitively intact individuals: A cross-sectional case-control study. BMC Geriatr..

[7] Ahn S., Chung J.W., Crouter S.E., Lee J.A., Lee C.E., Anderson J.G. (2023). Gait and/or balance disturbances associated with Alzheimer’s dementia among older adults with amnestic mild cognitive impairment: A longitudinal observational study. J. Adv. Nurs..

[8] Kim Y.J., Park I., Choi H.C., Ahn M.E., Ryu O.H., Jang D., Lee U., Lee S.K. (2023). Relationship of Neural Correlates of Gait Characteristics and Cognitive Dysfunction in Patients with Mild Cognitive Impairment. J. Clin. Med..

[9] Lin R.H., Wang C.C., Tung C.W. (2022). A Machine Learning Classifier for Predicting Stable MCI Patients Using Gene Biomarkers. Int. J. Environ. Res. Public Health.

[10] Blanco K., Salcidua S., Orellana P., Sauma-Perez T., Leon T., Steinmetz L.C.L., Ibanez A., Duran-Aniotz C., de la Cruz R. (2023). Systematic review: Fluid biomarkers and machine learning methods to improve the diagnosis from mild cognitive impairment to Alzheimer’s disease. Alzheimer’s Res. Ther..

[11] Wen J., Varol E., Yang Z., Hwang G., Dwyer D., Kazerooni A.F., Lalousis P.A., Davatzikos C., Colliot O. (2023). Subtyping Brain Diseases from Imaging Data. Machine Learning for Brain Disorders.

[12] Frizzell T.O., Glashutter M., Liu C.C., Zeng A., Pan D., Hajra S.G., D’Arcy R.C.N., Song X. (2022). Artificial intelligence in brain MRI analysis of Alzheimer’s disease over the past 12 years: A systematic review. Ageing Res. Rev..

[13] Lu W., Zhang M., Yu W., Kuang W., Chen L., Zhang W., Yu J., Lu Y. (2023). Differentiating Alzheimer’s disease from mild cognitive impairment: A quick screening tool based on machine learning. BMJ Open.

[14] Adelson R.P., Garikipati A., Maharjan J., Ciobanu M., Barnes G., Singh N.P., Dinenno F.A., Mao Q., Das R. (2023). Machine Learning Approach for Improved Longitudinal Prediction of Progression from Mild Cognitive Impairment to Alzheimer’s Disease. Diagnostics.

[15] Rykov Y.G., Patterson M.D., Gangwar B.A., Jabar S.B., Leonardo J., Ng K.P., Kandiah N. (2024). Predicting cognitive scores from wearable-based digital physiological features using machine learning: Data from a clinical trial in mild cognitive impairment. BMC Med..

[16] Chen P.H., Lien C.W., Wu W.C., Lee L.S., Shaw J.S. (2020). Gait-Based Machine Learning for Classifying Patients with Different Types of Mild Cognitive Impairment. J. Med. Syst..

[17] Fedorov A., Beichel R., Kalpathy-Cramer J., Finet J., Fillion-Robin J.C., Pujol S., Bauer C., Jennings D., Fennessy F., Sonka M. (2012). 3D Slicer as an image computing platform for the Quantitative Imaging Network. Magn. Reson. Imaging.

[18] Dale A.M., Fischl B., Sereno M.I. (1999). Cortical surface-based analysis. I. Segmentation and surface reconstruction. Neuroimage.

[19] Bahureksa L., Najafi B., Saleh A., Sabbagh M., Coon D., Mohler M.J., Schwenk M. (2017). The Impact of Mild Cognitive Impairment on Gait and Balance: A Systematic Review and Meta-Analysis of Studies Using Instrumented Assessment. Gerontology.

[20] Buracchio T., Dodge H.H., Howieson D., Wasserman D., Kaye J. (2010). The trajectory of gait speed preceding mild cognitive impairment. Arch. Neurol..

[21] Watson N.L., Rosano C., Boudreau R.M., Simonsick E.M., Ferrucci L., Sutton-Tyrrell K., Hardy S.E., Atkinson H.H., Yaffe K., Satterfield S. (2010). Executive function, memory, and gait speed decline in well-functioning older adults. J. Gerontol. A Biol. Sci. Med. Sci..

[22] Poulakis K., Pereira J.B., Mecocci P., Vellas B., Tsolaki M., Kloszewska I., Soininen H., Lovestone S., Simmons A., Wahlund L.O. (2018). Heterogeneous patterns of brain atrophy in Alzheimer’s disease. Neurobiol. Aging.

[23] van de Mortel L.A., Thomas R.M., van Wingen G.A., Alzheimer’s Disease Neuroimaging I. (2021). Grey Matter Loss at Different Stages of Cognitive Decline: A Role for the Thalamus in Developing Alzheimer’s Disease. J. Alzheimers Dis..

[24] Henstridge C.M., Hyman B.T., Spires-Jones T.L. (2019). Beyond the neuron-cellular interactions early in Alzheimer disease pathogenesis. Nat. Rev. Neurosci..

[25] Rokem A., Takemura H., Bock A.S., Scherf K.S., Behrmann M., Wandell B.A., Fine I., Bridge H., Pestilli F. (2017). The visual white matter: The application of diffusion MRI and fiber tractography to vision science. J. Vis..

[26] Wang Y., West J.D., Flashman L.A., Wishart H.A., Santulli R.B., Rabin L.A., Pare N., Arfanakis K., Saykin A.J. (2012). Selective changes in white matter integrity in MCI and older adults with cognitive complaints. Biochim. Biophys. Acta.

[27] Zhang H., Sachdev P.S., Wen W., Kochan N.A., Crawford J.D., Brodaty H., Slavin M.J., Reppermund S., Draper B., Zhu W. (2012). Gray matter atrophy patterns of mild cognitive impairment subtypes. J. Neurol. Sci..

[28] Loewenstein D.A., Acevedo A., Potter E., Schinka J.A., Raj A., Greig M.T., Agron J., Barker W.W., Wu Y., Small B. (2009). Severity of medial temporal atrophy and amnestic mild cognitive impairment: Selecting type and number of memory tests. Am. J. Geriatr. Psychiatry.

[29] Femir-Gurtuna B., Kurt E., Ulasoglu-Yildiz C., Bayram A., Yildirim E., Soncu-Buyukiscan E., Bilgic B. (2020). White-matter changes in early and late stages of mild cognitive impairment. J. Clin. Neurosci..

[30] Li W.X., Yuan J., Han F., Zhou L.X., Ni J., Yao M., Zhang S.Y., Jin Z.Y., Cui L.Y., Zhai F.F. (2023). White matter and gray matter changes related to cognition in community populations. Front. Aging Neurosci..

[31] Byun S., Han J.W., Kim T.H., Kim K., Kim T.H., Park J.Y., Suh S.W., Seo J.Y., So Y., Lee K.H. (2018). Gait Variability Can Predict the Risk of Cognitive Decline in Cognitively Normal Older People. Dement. Geriatr. Cogn. Disord..

[32] Du S., Ma X., Wang J., Mi Y., Zhang J., Du C., Li X., Tan H., Liang C., Yang T. (2023). Spatiotemporal gait parameter fluctuations in older adults affected by mild cognitive impairment: Comparisons among three cognitive dual-task tests. BMC Geriatr..

[33] Savica R., Wennberg A.M., Hagen C., Edwards K., Roberts R.O., Hollman J.H., Knopman D.S., Boeve B.F., Machulda M.M., Petersen R.C. (2017). Comparison of Gait Parameters for Predicting Cognitive Decline: The Mayo Clinic Study of Aging. J. Alzheimer’s Dis..

[34] Martin K.L., Blizzard L., Wood A.G., Srikanth V., Thomson R., Sanders L.M., Callisaya M.L. (2013). Cognitive function, gait, and gait variability in older people: A population-based study. J. Gerontol. A Biol. Sci. Med. Sci..

[35] Cai S., Chong T., Zhang Y., Li J., von Deneen K.M., Ren J., Dong M., Huang L., Alzheimer’s Disease Neuroimaging I. (2015). Altered Functional Connectivity of Fusiform Gyrus in Subjects with Amnestic Mild Cognitive Impairment: A Resting-State fMRI Study. Front. Hum. Neurosci..

[36] Schroeter M.L., Stein T., Maslowski N., Neumann J. (2009). Neural correlates of Alzheimer’s disease and mild cognitive impairment: A systematic and quantitative meta-analysis involving 1351 patients. Neuroimage.

[37] Vallar G., Di Betta A.M., Silveri M.C. (1997). The phonological short-term store-rehearsal system: Patterns of impairment and neural correlates. Neuropsychologia.

[38] Hanggi J., Streffer J., Jancke L., Hock C. (2011). Volumes of lateral temporal and parietal structures distinguish between healthy aging, mild cognitive impairment, and Alzheimer’s disease. J. Alzheimer’s Dis..

[39] Tullberg M., Fletcher E., DeCarli C., Mungas D., Reed B.R., Harvey D.J., Weiner M.W., Chui H.C., Jagust W.J. (2004). White matter lesions impair frontal lobe function regardless of their location. Neurology.

[40] McGough E.L., Kelly V.E., Logsdon R.G., McCurry S.M., Cochrane B.B., Engel J.M., Teri L. (2011). Associations between physical performance and executive function in older adults with mild cognitive impairment: Gait speed and the timed “up & go” test. Phys. Ther..

