# Supplementary material for: Machine Learning Model for Mild Cognitive Impairment Stage Based on Gait and MRI Images
Source: Brain Sci. 2024 May 9;14(5):480. doi: 10.3390/brainsci14050480 (PMC11119859; doi:10.3390/brainsci14050480)
Supplement: Supplementary file 1 [file brainsci-14-00480-s001.zip › brainsci-2986940-supplementary.pdf]

Supplement Table S1. The CNN architecture used for N (=20, 40, or 60) reduced feature dimensions in this study

	Layer Type	Activation Unit	Number of Filters	Kernel Size	Number of Stride	Padding	Output Size	Number of Parameters
**1**	Input						N X 1	
**2**	Conv1D	Relu	64	3	1	VALID	38 X 64	256
**3**	Conv1D	Relu	64	3	1	VALID	36 X 64	12352
**4**	Dropout (0.5)						36 X 64	0
**5**	MaxPooling1D	-	2	-	-	-	18 X 64	0
**6**	Flaten						1152	0
**7**	Fully connected	Relu					100	115300
**8**	Output	Sigmoid					2	202

Table S2. Performances of Gait dataset.

Algorithm for classification	Algorithm for feature reduction	No. of Features reduced	AUC	ACC	Recall	Precision	F1
**SVM**	**ICA**	**20**	0.61±0.05	0.67±0.06	0.71±0.07	0.93±0.08	0.78±0.05
		40	0.61±0.12	0.68±0.05	0.7±0.07	0.93±0.1	0.78±0.04
		60	0.51±0.06	0.62±0	0.64±0.13	0.88±0.09	0.77±0.02
	**PCA**	20	0.69±0.09	0.72±0.08	0.77±0.08	0.99±0.03	0.78±0.07
		40	0.65±0.1	0.7±0.09	0.73±0.07	0.99±0.03	0.78±0.07
		60	0.65±0.1	0.7±0.09	0.73±0.07	0.99±0.03	0.78±0.07
	**RP**	20	0.62±0.1	0.7±0.07	0.7±0.07	0.96±0.07	0.8±0.04
		40	0.67±0.09	0.71±0.09	0.76±0.1	0.97±0.05	0.79±0.05
		60	0.64±0.1	0.69±0.09	0.73±0.09	0.94±0.07	0.78±0.03
**RF**	**ICA**	**20**	0.51±0.08	0.59±0.08	0.63±0.05	0.86±0.13	0.72±0.07
		40	0.61±0.12	0.67±0.1	0.7±0.08	0.97±0.05	0.78±0.04
		60	0.58±0.07	0.64±0.07	0.68±0.07	0.88±0.12	0.75±0.06
	**PCA**	20	0.65±0.1	0.71±0.09	0.72±0.08	0.92±0.07	0.8±0.06
		40	0.65±0.07	0.71±0.1	0.71±0.08	0.95±0.08	0.8±0.06
		60	0.55±0.06	0.64±0.06	0.65±0.04	0.97±0.06	0.77±0.04
	**RP**	20	0.64±0.1	0.69±0.08	0.73±0.09	0.83±0.13	0.77±0.06
		40	0.62±0.11	0.67±0.09	0.72±0.09	0.82±0.14	0.75±0.08
		60	0.69±0.1	0.72±0.09	0.76±0.08	0.86±0.1	0.8±0.06
**CNN**	**ICA**	20	0.84±0.17	0.86±0.16	0.88±0.14	0.92±0.11	0.9±0.11

	40	0.95±0.09	0.97±0.08	0.97±0.08	0.99±0.03	0.98±0.05
	60	0.85±0.18	0.87±0.16	0.9±0.14	0.9±0.11	0.9±0.12
**PCA**	20	0.91±0.13	0.93±0.12	0.93±0.11	0.99±0.03	0.95±0.09
	40	0.93±0.12	0.95±0.1	0.94±0.1	0.99±0.03	0.96±0.07
	60	0.94±0.11	0.96±0.09	0.95±0.09	0.99±0.03	0.97±0.06
**RP**	20	0.95±0.09	0.96±0.08	0.96±0.09	0.99±0.03	0.97±0.06
	40	0.98±0.04	0.99±0.04	0.99±0.03	0.99±0.03	0.99±0.04
	60	0.94±0.1	0.95±0.1	0.96±0.08	0.97±0.06	0.96±0.08

Table S3. Performances of MRI dataset.

Algorithm for classification	Algorithm for feature reduction	No. of Features reduced	AUC	ACC	Recall	Precision	F1
**SVM**	**ICA**	20	0.62±0.1	0.65±0.1	0.72±0.11	0.99±0.03	0.77±0.01
		40	0.59±0.08	0.64±0.08	0.71±0.09	0.95±0.05	0.77±0.01
		60	0.5±0.12	0.62±0	0.62±0.11	0.87±0.1	0.77±0.01
	**PCA**	20	0.64±0.1	0.67±0.09	0.74±0.08	0.99±0.03	0.77±0.01
		40	0.64±0.1	0.67±0.1	0.74±0.1	0.97±0.05	0.77±0.01
		60	0.61±0.09	0.66±0.09	0.7±0.09	0.98±0.04	0.77±0.02
	**RP**	20	0.53±0.09	0.62±0	0.65±0.09	0.98±0.06	0.77±0.02
		40	0.64±0.1	0.66±0.1	0.75±0.09	0.97±0.05	0.77±0.02
		60	0.61±0.12	0.66±0.09	0.71±0.1	0.99±0.03	0.77±0.03
**RF**	**ICA**	**20**	0.56±0.08	0.63±0.07	0.67±0.05	0.85±0.12	0.73±0.07
		40	0.55±0.1	0.64±0.07	0.66±0.06	0.92±0.07	0.76±0.05
		60	0.54±0.08	0.63±0.06	0.65±0.05	0.92±0.09	0.75±0.04
	**PCA**	20	0.53±0.1	0.6±0.08	0.65±0.07	0.82±0.14	0.71±0.07
		40	0.59±0.05	0.67±0.05	0.68±0.04	0.93±0.1	0.78±0.03
		60	0.51±0.07	0.62±0.06	0.63±0.04	0.98±0.04	0.77±0.02
	**RP**	20	0.57±0.09	0.64±0.1	0.66±0.06	0.84±0.14	0.74±0.08
		40	0.56±0.09	0.62±0.08	0.67±0.06	0.81±0.14	0.73±0.06
		60	0.59±0.14	0.64±0.11	0.7±0.12	0.83±0.13	0.74±0.08
**CNN**	**ICA**	20	0.84±0.13	0.84±0.13	0.91±0.1	0.83±0.14	0.86±0.12

	40	0.9±0.1	0.92±0.07	0.93±0.1	0.96±0.05	0.94±0.05
	60	0.84±0.21	0.86±0.19	0.88±0.17	0.94±0.12	0.9±0.14
**PCA**	20	0.94±0.1	0.96±0.08	0.95±0.09	0.99±0.03	0.97±0.05
	40	0.93±0.13	0.94±0.12	0.95±0.1	0.97±0.06	0.96±0.08
	60	0.93±0.13	0.94±0.11	0.95±0.11	0.98±0.04	0.96±0.08
**RP**	20	0.91±0.16	0.92±0.17	0.93±0.14	0.96±0.09	0.95±0.1
	40	0.9±0.14	0.92±0.12	0.92±0.12	0.98±0.04	0.95±0.08
	60	0.93±0.12	0.95±0.1	0.94±0.11	0.99±0.03	0.96±0.07

Table S4. Performances of Gait + MRI dataset.

Algorithm for classification	Algorithm for feature reduction	No. of Features reduced	AUC	ACC	Recall	Precision	F1
**SVM**	**ICA**	20	0.65±0.1	0.68±0.1	0.75±0.1	0.84±0.11	0.77±0.03
		40	0.59±0.09	0.64±0.09	0.69±0.08	0.95±0.05	0.77±0.03
		60	0.5±0	0.62±0	0.62±0.11	0.84±0.1	0.77±0
	**PCA**	20	0.66±0.1	0.68±0.1	0.78±0.09	0.99±0.03	0.77±0.01
		40	0.64±0.09	0.66±0.1	0.75±0.09	0.94±0.09	0.77±0.01
		60	0.61±0.08	0.64±0.08	0.71±0.08	0.97±0.05	0.77±0.01
	**RP**	20	0.61±0.15	0.62±0.14	0.72±0.14	0.94±0.1	0.77±0.02
		40	0.65±0.14	0.68±0.14	0.73±0.11	0.99±0.03	0.77±0.01
		60	0.61±0.08	0.67±0.03	0.72±0.07	0.96±0.07	0.78±0.02
**RF**	**ICA**	**20**	0.57±0.11	0.62±0.12	0.67±0.07	0.86±0.07	0.74±0.04
		40	0.51±0.11	0.59±0.1	0.63±0.07	0.86±0.17	0.72±0.1
		60	0.5±0.05	0.59±0.06	0.63±0.03	0.84±0.16	0.71±0.08
	**PCA**	20	0.62±0.06	0.68±0.08	0.7±0.05	0.88±0.12	0.77±0.07
		40	0.6±0.08	0.66±0.08	0.68±0.04	0.92±0.06	0.77±0.04
		60	0.56±0.07	0.65±0.06	0.66±0.05	0.96±0.05	0.77±0.05
	**RP**	20	0.54±0.1	0.62±0.1	0.65±0.06	0.85±0.14	0.73±0.09
		40	0.57±0.11	0.64±0.1	0.67±0.08	0.86±0.08	0.75±0.06
		60	0.61±0.1	0.66±0.11	0.69±0.06	0.86±0.11	0.75±0.09
**CNN**	**ICA**	20	0.86±0.17	0.89±0.16	0.89±0.14	0.96±0.05	0.92±0.11

	40	0.87±0.15	0.88±0.13	0.93±0.13	0.91±0.08	0.91±0.09
	60	0.9±0.14	0.91±0.13	0.94±0.11	0.91±0.1	0.92±0.1
**PCA**	20	0.9±0.12	0.91±0.12	0.94±0.09	0.93±0.06	0.93±0.1
	40	0.93±0.11	0.94±0.11	0.95±0.09	0.96±0.09	0.95±0.09
	60	0.93±0.11	0.94±0.11	0.95±0.09	0.97±0.05	0.95±0.09
**RP**	20	0.93±0.12	0.95±0.1	0.94±0.11	0.99±0.03	0.96±0.07
	40	0.92±0.15	0.94±0.12	0.94±0.14	0.99±0.03	0.96±0.08
	60	0.94±0.1	0.95±0.1	0.95±0.08	0.98±0.04	0.96±0.07
